# ﻿Current use of holy mushrooms of the genus *Psilocybe* in a Zapotec community in Oaxaca, Mexico

**DOI:** 10.3897/imafungus.16.148070

**Published:** 2025-05-23

**Authors:** Mara Ximena Haro-Luna, Felipe Ruan-Soto, Virginia Ramírez-Cruz, Julieta Amaya-Pérez, Laura Guzmán-Dávalos

**Affiliations:** 1 Departamento de Botánica y Zoología, Universidad de Guadalajara, Apdo. postal 1–139, 45147, Zapopan, Jalisco, Mexico Universidad de Guadalajara Zapopan Mexico; 2 Laboratorio de Procesos Bioculturales, Educación y Sustentabilidad, Instituto de Ciencias Biológicas, Universidad de Ciencias y Artes de Chiapas, Libramiento Norte Pte., Caleras Maciel, 29000 Tuxtla Gutiérrez, Chiapas, Mexico Universidad de Ciencias y Artes de Chiapas Tuxtla Gutiérrez Mexico; 3 San Miguel Mixtepec, Oaxaca, Mexico Unaffiliated San Miguel Mixtepec Mexico

**Keywords:** Ceremonial use, entheogens, ethnomycology, ritual practices, traditional knowledge

## Abstract

The use of psychoactive *Psilocybe* mushrooms as entheogens by the Mazatecs of Oaxaca became known to the world in 1957. While the Mazatec Region has been the focus of research, historical records indicate that other indigenous groups in Mexico, including the Zapotecs, also used these mushrooms for ceremonial and medicinal purposes. However, the linguistic, cultural and ecological diversity of the Zapotec people suggests that their practices cannot be generalised. In contemporary times, changes in cultural and environmental factors, as well as the rise of psychedelic tourism, have contributed to the transformation and commodification of these traditions. The purpose of this paper was to document the changes in the use, customs and knowledge of *Psilocybe* species in a Zapotec community in the Valles Centrales of Oaxaca. Through informal in–depth interviews, 30 people from the community of El Peral, San Antonino El Alto were interviewed. These testimonies were recorded in a field diary and entered into a database for categorical analysis. In this way, it was possible to document that the use of *Psilocybezapotecorum*, called Hongo Borracho or Hongo Santo and in Zapotec Ni’to be’ya, for healing and divinatory purposes, continues in the community. However, its use is decreasing and the mushrooms are more difficult to find, likely due to changing climatic patterns, according to those interviewed. We found that there are still people dedicated to the sale of these mushrooms. For the Zapotecs of El Peral, these mushrooms can do whatever is asked of them according to a ritual, but they are aware that outsiders used them for recreational purposes, although they did not oppose it. This study underscores the importance of documenting and understanding cultural practices related to mushrooms, as well as the need to address environmental challenges that affect their availability and traditional use. Finally, this is the first formal record of the use of *Psilocybe* mushrooms amongst Zapotecs of the Valles Centrales Region in Oaxaca.

## ﻿Introduction

The traditional use of holy mushrooms of the genus *Psilocybe* in the Mazatec culture was published in 1957 by R. Gordon Wasson in Life magazine (Wasson 1957a) and, in the same year, in a less known article, the experience of eating the mushrooms by Valentina Pavlova Wasson and her daughter in This Week Magazine (Wasson 1957b; Williams et al. 2020). Although the Life magazine article originally omitted the name of the Mazatec town, Huautla de Jiménez, where this author participated in a mushroom ceremony, the publicity generated by this discovery led to people interested in trying these mushrooms to find the location (Siff 2018). As a result, their ceremonial use was displaced, allowing the spread of recreational use, which purists of ritual entheogen consumption considered “vulgarised, profane, childish and largely hedonistic” (Letcher 2007). In this context, the cultural significance is neither understood nor respected (Estrada Martínez et al. 2016). Currently, according to Johnstad (2018), the use of these mushrooms is booming amongst modern Westerners as a way to compensate for the lack of spiritual experiences in contemporary lifestyles. For some Mazatec people, this is beneficial since they make a living from it, but for those who continue to use them traditionally, it represents a lack of respect associated with this phenomenon (Estrada Martínez et al. 2016).

Although the study of the use of *Psilocybe* mushrooms for medicinal or divinatory purposes has received the most attention in the Mazatec Region, their use was also documented in other Mexican cultures, such as the Nahuas and Matlatzincas in central Mexico and the Chatinos, Chinantecs, Mixes, Mixtecs and Zapotecs in the State of Oaxaca (Ramírez-Cruz et al. 2006; Guzmán 2008, 2014; Spiers et al. 2024). In the Oaxacan groups, the use of nine *Psilocybe* species has been reported, five of them being used by the Zapotecs from Sierra Sur Region (Guzmán 1959, 2012; Ramírez-Cruz et al. 2006; Cortés-Pérez et al. 2021). Specifically, studies with the Zapotecs were carried out in San Agustín Loxicha by Heim in 1957 (Singer and Smith 1958; Guzmán 1959). Recently, there has been research on the “psychedelic tourism” that has been attracted to San José del Pacífico since 1957, which has changed the identity of the place to offer the experience sought by visitors with hallucinogenic mushrooms, leaving traditions aside (Lariagon 2020).

Although there have been previous records of the ceremonial use of mushrooms of the genus *Psilocybe* by the Zapotecs, for instance, in San Agustín Loxicha, located in the Sierra Sur Region, as mentioned above, or in San Miguel Mixtepec, located in the Valles Centrales (Amaya Pérez 2020), these findings were particular to the Zapotecs of certain regions and the information was scarce. It is known that Zapotecs is the largest and most numerous indigenous group in the State of Oaxaca, Mexico. They call themselves Binnizá, which means “people who come from the clouds” (Gómez 2006). Their territory encompasses five geographical regions: Coastal Region, Isthmus of Tehuantepec, Sierra Norte, Sierra Sur and Valles Centrales (Aparicio 2008). This great geographical diversity and, therefore, the ecological diversity, in which the Zapotec people have settled, is reflected in their linguistic and cultural diversity. Zapotec belongs to the Otomanguean linguistic family, the largest and most diverse in Mexico. There are currently 62 variants of Zapotec (INALI 2024), within which there are interregional and even interdistrict differences. Therefore, it is not possible to speak of Zapotec culture in a unitary way. In general, the Zapotecs can be divided into four macro–ethnicities based on their geographical location: Zapotecs from the Isthmus, from the Sierra Norte, from the Sierra Sur and from the Valles Centrales, the latter being the oldest group (Filgueiras 2020).

Even though Zapotecs today are mostly Catholic, rituals related to nature and meteorological phenomena are still performed in the communities (Sánchez-Antonio 2022). In addition, there is a strong worship of water; for example, rituals are performed in which the Rayo (thunderbolt as a deity) is asked for wellness and health in springs, wetlands and caves, known as casas de Rayo (thunderbolt houses) (González Pérez 2016; Filgueiras 2020). In the Zapotec cosmovision, illness is caused by an individual imbalance and health is perceived as a state of integral harmony that involves the balance of the body, as well as relationships with other people and the natural and supernatural environment (Aparicio 2008). According to Guzmán (1959), for some indigenous groups in Oaxaca, such as the Zapotecs, one way to address personal or community problems or ailments is through the ingestion of hallucinogenic mushrooms in nocturnal ceremonies.

Although traditions and ceremonies have persisted amongst indigenous groups in Oaxaca since the Spanish conquest, it is not known whether the ritual use of *Psilocybe* mushrooms reported in the 1950s is still in effect, has changed or has disappeared. This is because it has been shown that the introduction of hegemonic cultures, as well as environmental, cultural and lifestyle changes have caused a loss of the traditional knowledge (e.g. Haro-Luna et al. (2022a) in Jalisco State). This deterioration is observed in the local knowledge of the traditional medicine, since this knowledge is no longer considered useful for the new sociocultural conditions (Reyes-García et al. 2013; Arjona-García et al. 2021). On the other hand, in recent times, these mushrooms have gained significant economic importance in locations such as Huautla de Jiménez and San José del Pacífico, where the healing ritual has been re-interpreted and transformed into a commodity with monetary value and as a tourist attraction (Spitalier 2023). This has led to a debate about the exploitation of this resource, as well as with other psychedelics (Horák 2018) and about the misrepresentation of the spirituality assumed by Westerners when consuming these mushrooms.

Therefore, the objectives of this work are: 1) to document the current traditional use of mushrooms of the genus *Psilocybe* in a Zapotec community of the Valles Centrales; 2) to identify the species used; 3) to describe the cosmovision, beliefs, methods of use, local names, sale and other data related to these mushroom species amongst the people of the community of El Peral, Municipality of San Antonino El Alto, Oaxaca, Mexico and, above all, 4) to study the people’s perception of the loss of this knowledge and 5) their perception of the use of these mushrooms by people outside their community.

## ﻿Materials and methods

### ﻿Study area

**Figure 1.**
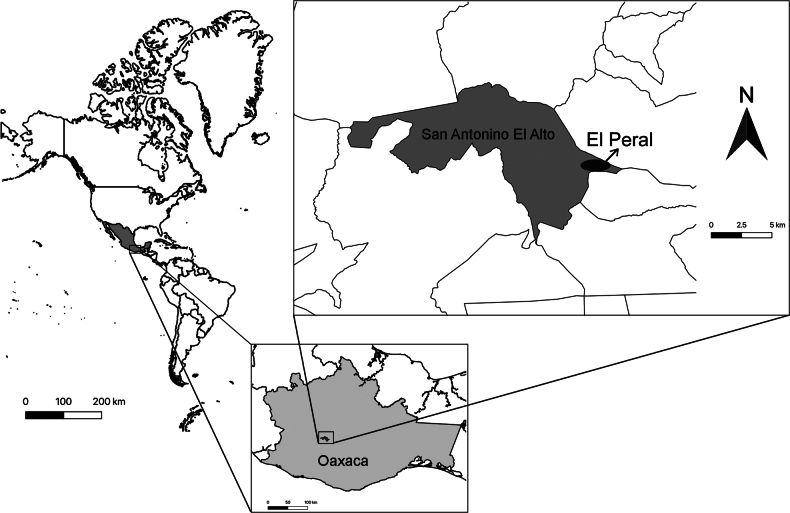
Map of El Peral community, municipality of San Antonino El Alto, Valles Centrales, Zapotec Region, Oaxaca, Mexico.

The community of El Peral is located at the coordinates 16°49'15"N, 96°57'51"W and belongs to the Municipality of San Antonino El Alto, Oaxaca, Mexico (Fig. 1). This Municipality covers an area of 60.07 km^2^, had 14 communities with a total population of 2,705, of which 42.2% had completed primary education, 34.6% had completed secondary education, 20.55% had completed high school and only 1.92% hold a college degree. The illiteracy rate in San Antonino El Alto was 10.2% in 2020. Of the total illiterate population, 24.4% were men and 75.6% were women. Additionally, only 22.5% of households have access to the internet. Almost 58.7% of the population was economically active. The 37.95% of the population aged three years and older speaks an indigenous language, in this case Zapotec (INEGI 2020). The loss of the Zapotec language may be affecting the transmission of traditional ecological knowledge about biodiversity, as language is a fundamental vehicle for cosmovision and cultural practices (Arriaga-Jiménez et al. 2018).

El Peral is located at an elevation of 2,550 m a.s.l. and has a predominantly temperate climate with an average annual temperature between 12 and 18 °C, a range of -3 to 24 °C and an average annual rainfall of 200 to 1800 mm. A total of 72% of its territory is composed of pine forests, with species such as *Pinuspatula* and *Pinusoocarpa*, 26% being used for agriculture and only 2% are oak forests (*Quercus* spp.). By 2020, it had a population of 161 inhabitants, of which 80 were women and 81 men. A total of 88.2% of its population identified themselves as Zapotec (INEGI 2020; CONABIO 2024). The primary economic activity of the inhabitants was agriculture. They grew vegetables, ornamental flowers and fruits such as peaches, apples and blackberries (INEGI 2020).

### ﻿Data collection

The fieldwork was carried out from June 2021 to May 2024. In the first phase, the motives and objectives of the research were explained and permission was obtained from the traditional authorities of El Peral, as well as from each of the people involved in this work. This permission was renewed each year, according to the changes in the positions of the traditional leaders, following the code of ethics of the Latin American Society of Ethnobiology (Cano-Contreras et al. 2016). As part of this process, initial contact was made with the traditional authority of the community, where the purpose of the study and the intention to publish the findings were explained. Additionally, we enquired about possible ways to provide a form of retribution. The acting community leader convened a general assembly, where the project was discussed with all community members. Three days later, we were informed that there was a collective agreement to proceed. Furthermore, during each visit and interview, we reiterated the purpose and scope of the study, as well as the possibility of publication and the logistical aspects involved, to which each interviewee expressed their consent. When the community leadership changed, we once again sought permission and a new assembly was convened. On this occasion, they collectively decided to request a mushroom cultivation workshop as a form of retribution, which we provided in 2023 for those interested.

Additionally, co-author Julieta Amaya-Pérez (JAP), despite being originally from the neighbouring community of San Miguel Mixtepec, served as the local collaborator. Beyond her role as a translator, biologist and key contact, she has been a pre-school teacher in El Peral for over ten years and has resided there during this time.

This study adopted an ethnographic approach, emphasising the local context and prioritising the perspectives of the participants. Informal in–depth interviews were conducted with 30 individuals between 19 and 95 years old, distributed as follows: two participants aged 19–21, two aged 25–34, eight aged 35–44, six aged 45–54, five aged 55–64, three aged 65–74 and two aged 75–84. Additionally, there were two participants over 85 years old. This distribution ensured a broad representation of different age groups within the community. These interviews were notably easy to carry out due to the openness of the participants in discussing the topic. In fact, upon simply mentioning the objectives of the study, many eagerly began sharing their knowledge with a sense of pride. This information was recorded in the field diary and entered into a database for categorical analysis (Echeverría 2005). The quality collaborators were selected using the snowball method (Noy 2006), recognised by local people for using or selling mushrooms of the *Psilocybe* genus to cure various illnesses. Through direct observation (Fink 1955), the processes of mushrooms harvest and consumption were described by three people who agreed to be observed. In addition, ethnographic data on the relationship between adolescents and mushrooms, as well as the interaction processes and their perceptions, were also obtained through direct observation.

Three ethnomycological expeditions or walk–in–the–woods (Zent and Zent 2010) were carried out, accompanied by two local experts, to collect the specimens of mushrooms used for these purposes; however, specimens were only found during one trip in July 2023. The lack of findings in previous years was likely due to unfavourable climatic conditions present during our visits. Given the lack of fresh specimens in those years, community members shared with us dried mushrooms they had stored from previous years, as well as photographs from their mobile phones, allowing us to verify to which species they corresponded. The specimens collected in 2023 were described fresh and then dehydrated for transport and finally deposited in the Mycological Collection of the Dr. Luz María Villarreal de Puga Herbarium of the Institute of Botany of the University of Guadalajara (IBUG), as M.X. Haro-Luna 492, 493 and 494. The specimens were studied using conventional mycological techniques, based on macroscopic and microscopic characteristics (Largent et al. 1977; Largent 1981).

## ﻿Results and discussion

The three specimens collected during the ethnomycological surveys, as well as those shown by residents, both fresh and dried, were determined as *Psilocybezapotecorum* according to Guzmán (1983, 1995, 2012). Therefore, it was found that people from this locality use only one species of hallucinogenic mushrooms, *P.zapotecorum*. It is important to highlight that participants consistently showed us their mushrooms and photographs, confirming that they were always the same species in all cases. It is also worth noting that sometimes people dry the mushrooms for storage and that these specimens further allowed us to confirm the exclusive use of *P.zapotecorum* in the community.

According to the people interviewed in this work, there were few villagers who do not use or know these mushrooms. Those who did not know mushrooms were outsiders who came to the community because they married locals. Those who sold mushrooms were well known in the community, as were the older adults who explained how to consume *Psilocybe*. However, there was no specific cultural role associated with the use and sale of these mushrooms, as knowledge and consumption were present amongst people with different occupations, including bakers, merchants, homemakers and farmers.

### ﻿Local nomenclature

*Psilocybezapotecorum* had several names in El Peral. In Spanish, they were known as Hongo borracho (Drunken mushroom), as Fray Bernardino de Sahagún called them in the “Historia de las Cosas de la Nueva España” (Giménez 1988), because of the effect these mushrooms had on people, like drunkenness. However, older adults said that they were not drunken mushrooms, but Hongos Santos (Holy mushrooms), a name also used by María Sabina in Huautla de Jiménez, according to Estrada (1977). They were also known as Nanacatitos or Nanacates, which derive from a Nahuatl name from central Mexico (Schultes 1940; Reyes-López et al. 2020), due to the influence of the Nahuatl culture on the ancient Mixtecs and Zapotecs of Oaxaca; both words mean mushrooms and are generally used to refer flesh (Guzmán 2011).

As previously mentioned, Zapotec language has multiple variants. In El Peral, *Psilocybe* mushrooms were called Ni’to be’ya or Ni’to be meaning “Holy mushroom”, as was reported in San Miguel Mixtepec in the research of Amaya Pérez (2020). According to Garibay-Orijel (2009), in the Zapotec of Valles Centrales, Be’ya is a word that includes all mushrooms, although in other Zapotec regions, such as Sierra Norte, they are called Be neeche and Be ya yeri. On the other hand, Hunn et al. (2015) found that all mushrooms were called Měy by the Zapotecs of San Juan Mixtepec in Sierra Sur, which is very different from the Zapotec variant spoken in El Peral, which is closer to the variants of other localities in Valles Centrales and of Sierra Norte. Regardless of the terminology, when the people of El Peral talked about them, they always did so with respect and affection. Even those who said they had never eaten them or did not know them because they came from other villages, spoke of them with reverence and considered them medicine.

Adolescents no longer referred to these mushrooms in Zapotec, but simply called them “mushrooms” or “drunken mushrooms” in Spanish. Although local adolescents between 12 and 20 years old considered themselves Zapotecs and understood the language, they preferred not to speak it, which may be linked to a lack of interest in learning and understanding the ritual and traditional use of these mushrooms. A similar case has been reported amongst the Zapotec people of the Isthmus Region, where traditional ethnobotanical knowledge has been gradually lost due to young people’s disinterest in both their language and traditional knowledge (Saynes-Vásquez et al. 2013). Additionally, the presence of individuals from non-Zapotec communities has influenced local linguistic dynamics.

### ﻿Local taxonomy

**Figure 2.**
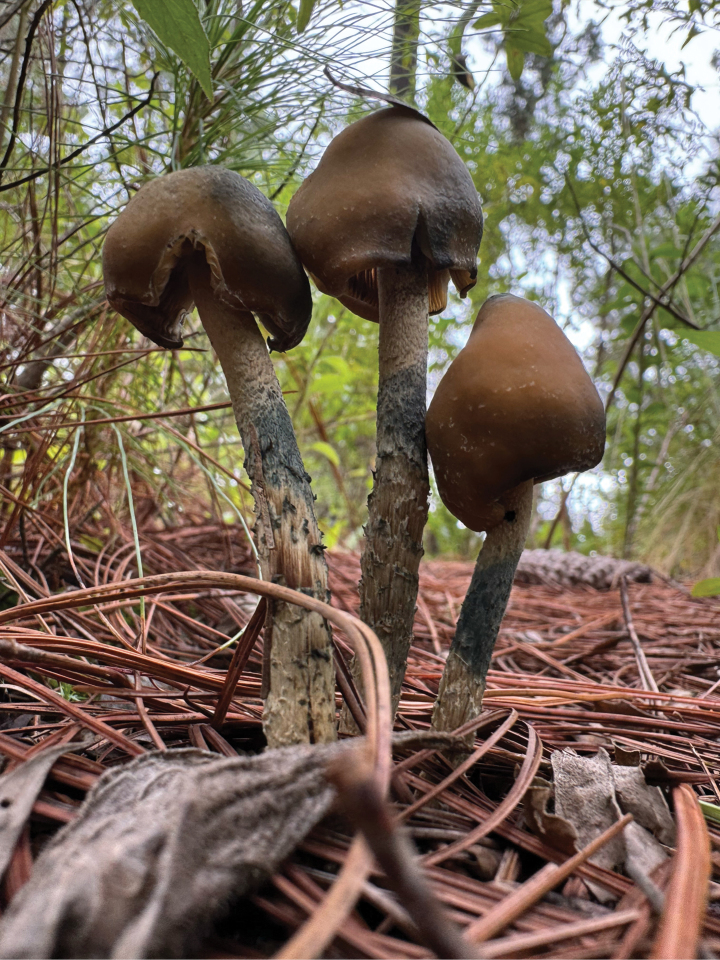
*Psilocybezapotecorum* mushrooms in the field, where the blue stain can be seen in the stipes after mistreatment.

People recognised Nanacatito or Hongo Santo by different morphological and organoleptic characteristics. For example, they said that “it is very similar to the *Nanacate de Cuero*” (leathery mushroom) (*Infundibulicybe* spp.), “but this one is tougher and more resistant” (Anonymous 3, female, 40 years old, 2022). In addition, in the case of the Nanacatito “when you squeeze the little foot, it turns purple or blue” (Anonymous 16, female, 92 years old, 2024). This bluish colouration in *Psilocybe* mushrooms is produced due to the action of a laccase and a phosphatase that degrades psilocin, forming a mixture of quinonoid oligomers responsible for the blue colour (Lenz et al. 2019). Although the Zapotecs mainly mentioned this change in colour (Fig. 2), it was common for them to recognise these mushrooms by their smell of damp earth. They mentioned that the cap has a characteristic “sucker”, referring to the umbonate shape of the pileus in mycological terminology, which is usually present in *Psilocybezapotecorum* (Guzmán 1983; Cortés-Pérez et al. 2021).

### ﻿Ecology

According to the interviewees, Hongos Santos were difficult to find. They considered that the most important thing was the substrate on which it grows. Therefore, it was common for them to mention that “the mushroom likes ugly places, like sandy or collapsed earth” (Anonymous 15, female, 87 years old, 2023). This was also reported by the Mazatecs, who named and described them as “little ones that sprout from the landslide” or mushrooms that grow in landslides or mudslides on roads (Ríos-García et al. 2022).

Regarding the seasonality, they mentioned that it was necessary that it rained for several days, “the Nanacatitos are born when it rains for three days or three nights” (Anonymous 3, female, 78 years old, 2022), between the months of July and August. However, they also do not bear fruit if there is too much humidity, which they illustrated as follows: “if it gets too wet or if the swamp turns green, the mushrooms don’t come out, they get embarrassed” (this is a way of expressing that they do not sprout because they are ashamed to go outside and remain underground) (Anonymous 2, female, 63 years old, 2022). It is worth noting that all interviewees and villagers pointed out that the climate has changed and “it doesn’t rain like it used to” (Anonymous 9, male, 50 years old, 2022). In people’s perceptions, this has made it much more difficult to find *Psilocybezapotecorum* and is one of the reasons why its use and trade has decreased significantly in the last five years. This correlation between rainfall and the biomass of wild mushrooms of different species collected has also been found in other continents, specifically in the Czechia, Austria, Great Britain and the Nordic countries (Procházka et al. 2023).

All interviewees emphasised that, in recent years, only a few people had managed to consume or even find Nanacatitos. They went out to look for them, but “there’s not much growing anymore because it doesn’t rain much” (Anonymous 15, female, 87 years old, 2023). This drought was attributed to deforestation, as they mentioned “there used to be more mushrooms, it used to rain more but people have deforested a lot and that’s why it doesn’t rain as much” (Anonymous 10, female, 45 years old, 2022). Nevertheless, they also mentioned that this scarcity of mushrooms was caused by climate change and overexploitation of resources, which would lead to a loss of traditional knowledge: “When we run out of mushrooms and they don’t grow anymore, there will be no one to look for them, so they won’t be used anymore” (Anonymous 16, female, 92 years old, 2024). This reflects the relationship between biodiversity and the way cultures use and manage these resources (Boege 2021). Similar situations are documented around the world regarding how climate change is negatively affecting local knowledge about biodiversity, especially medicinal plants and mushrooms, which have become harder to find for those who use them (Pearson et al. 2021). However, according to the precipitation records from CONAGUA (2024), although there have been years with periodic droughts, in El Peral, the average rainfall in recent years has decreased since 2020, but not significantly.

Regarding the quantity of mushrooms, they expressed that before, it was possible to collect larger quantities of mushrooms, but nowadays very small quantities are collected, which they also attribute to the recent lack of rain. On the other hand, they also mentioned that collecting them in large quantities is not bad because “they grow back” (Anonymous 2, female, 63 years old, 2022), as long as they are used in a good way. This is because there is a belief that mushrooms should not be collected if they are not going to be used because in addition to the lack of rain, these actions can also harm the abundance of Ni’to be’ya mushrooms in the coming years. This perception of the loss due to the disrespectful use of traditional norms has also been reported in South American cultures (Suárez 2012) and Tsotsil people from Chiapas, Mexico (Ruan-Soto 2018). Although this is far from being evidence of a sense of conservationist mindset, it is most often seen as a divine punishment. Estrada Martínez et al. (2016) also reported that, in the Mazatec Region, people feel that fewer mushrooms grow now because they have been overexploited for capital gain, without being thanked for having grown. Regarding the current scarcity of mushrooms in the forests, they also expressed that this occurs because the entire ecosystem is affected. “The *Nanacate* wants there to be animals and everything, but people destroy everything. That is why it is born where the squirrel and the deer pass” (Anonymous 5, female, 40 years old, 2022).

### ﻿Harvesting

The interviewees agreed that not everyone can find the Nanacatitos when they go out looking for them, even if they know the places where they usually grow and travel long distances within the forest. Even if the mushrooms are there, if it is not the time for that person to find them or if they are not supposed to find them, it will not be possible to see them. Even those who collect *Psilocybezapotecorum* mushrooms for sale sometimes fail to find specimens.

We were told that, in the past, people would go out to look for mushrooms on someone else’s orders or when they intended to sell them. Nowadays, however, it is more common for them to collect them opportunistically while carrying out their daily activities, such as gathering firewood, bathing in the river or taking care of their crops. This casual gathering has also been reported for edible fungi in southern and western Mexico (Ruan-Soto et al. 2006; Farfán-Heredia et al. 2018; Haro-Luna et al. 2022b), particularly in tropical forests and dry areas, where energy expenditure considerations often shape foraging practices (Ruan-Soto 2018; Haro-Luna et al. 2022b). However, in temperate forests, this behaviour is less common, especially when it involves species of high cultural or economic importance. In these cases, targeted harvest trips are more likely to occur (Montoya et al. 2008). For instance, in El Peral, it is common to send children to gather these mushrooms after school.

Once collected, it is customary to take five to six sporomes of *Psilocybezapotecorum*, which are wrapped in leaves of the San Pablo plant (*Wigandiaurens*), forming a package. In the case of large mushrooms, only three are placed on the leaf. Each package of mushrooms is considered a dose. This method of transportation and sale is also reported amongst the Mazatecs of Huautla de Jiménez, with the differences that these wrappings contain pairs of mushrooms, two or three pairs and of this and other *Psilocybe* species (García de Teresa 2022).

The people of El Peral mentioned that the mushrooms should not be collected by the sick person, but that someone else should be sent to look for and collect them. “If you get sick, you can’t cut the mushroom yourself, someone else has to give it to you” (Anonymous 12, female, 74 years old, 2023). Some gatherers even make the sign of the cross when they find mushrooms or before they cut them. This is done to prevent the person who is going to eat them from feeling bad and to thank God for the good fortune of finding them.

**Figure 3.**
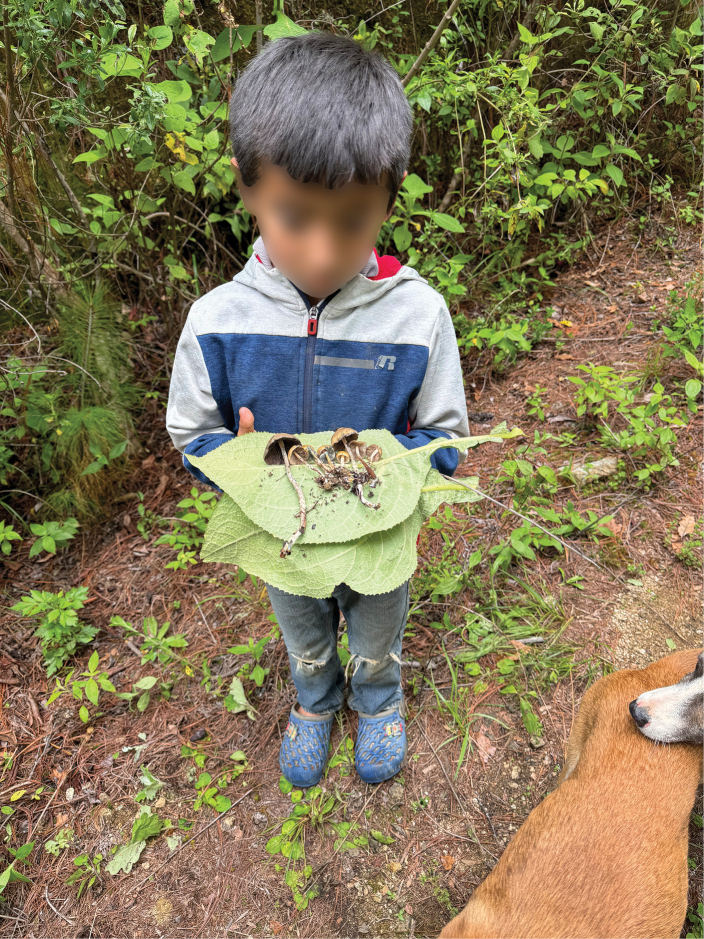
Child harvester of *Psilocybezapotecorum* from El Peral holding mushrooms on the leaves of the San Pablo plant.

The emotional state and age of the mushroom picker play a significant role in shaping the experiences and visions one will have after eating mushrooms. All interviewees said that mushrooms take on the personality of the person who picks them. “If a child cuts them, a child talks to you, if an adult cut them, an adult talk to you” (Gabriel, male, 30 years old, 2022) (Fig. 3). For example, if they are harvested by a rude and angry person, those who consume them will feel that someone with these characteristics is talking to them or may even have aggressive visions. “If an angry man cuts it, the Nanacatito will also be like that” (Anonymous 16, female, 92 years old, 2024). On the other hand, if the mushrooms are picked by serious children or adults, “the mushroom will not speak” (Anonymous 16, female, 92 years old, 2024), meaning that, while visual experiences may occur, the consumer will not hear the message or receive the answers they seek through the experience. In the context of El Peral, interviewees explained that they specifically prefer mushrooms picked by cheerful, talkative children, as mushrooms are then believed to embody these same traits — joyful, lively and communicative — during the trance, allowing consumers to receive clearer and more satisfying answers to their questions.

### ﻿Sale of mushrooms

**Figure 4.**
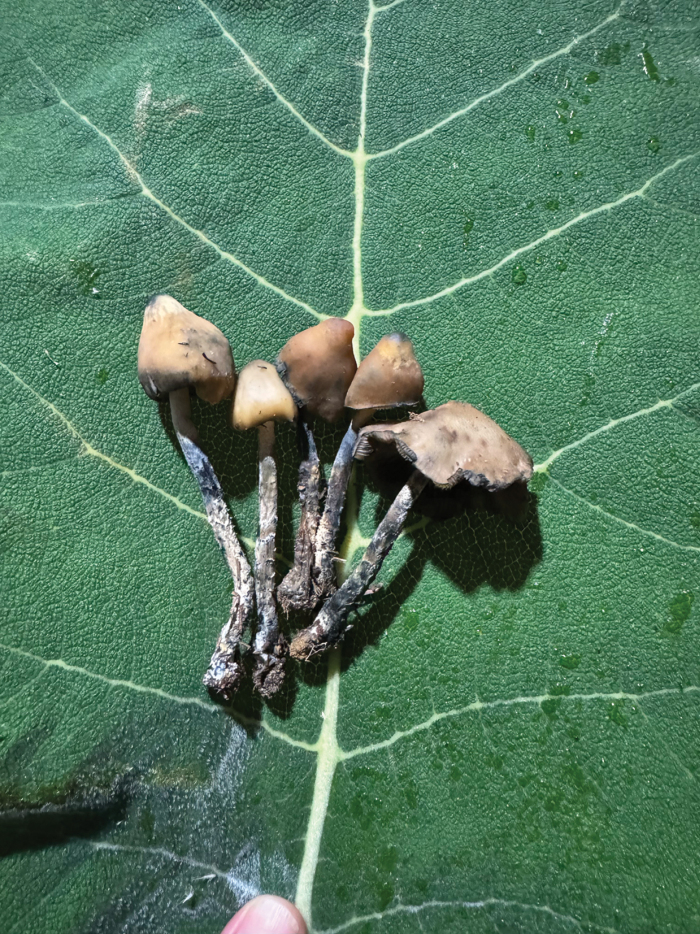
A dose of mushrooms on of a leaf of San Pablo plant, which will be sold in San Antonino.

The packages of mushrooms wrapped in leaves (Fig. 4) were sold within the community of El Peral or in the municipal capital of San Antonino, where a package, equivalent to a personal dose of mushrooms, costs about 25 Mexican pesos (about $1.5 US dollar), but when they were taken to neighbouring communities such as San Miguel Mixtepec, prices ranged from 100 to 200 pesos (around $5 to 10 dll). Unlike in Huautla (Rodríguez Venegas 2017; García de Teresa 2022), there were no intermediaries in El Peral. The pickers themselves were the ones who sold the mushrooms.

People pointed out that “it is not sold like it used to be; in the past it was sold a lot, now it is not sold because it is running out and now only older people believe that the Nanacatito is useful for healing” (Anonymous 16, female, 92 years old, 2024). They thought that young people no longer consume or buy mushrooms because they no longer believe in the divinatory or healing properties of mushrooms. Even the young interviewees, who were between 16 and 20 years old at the time of fieldwork, said they consumed mushrooms for the sensory experience, not for their healing properties or that they collected them to sell. This disinterest of the younger generation in preserving traditions has been widely documented in different regions of the world (e.g. Wagh and Jain (2015); Maleknia et al. (2024)) due to sociocultural changes and apathy towards their ethnic identity (Haro-Luna et al. 2022a).

The Zapotecs of El Peral mentioned that they had heard of people from San Pedro, Mixtepec, who kept dried mushrooms to sell throughout the year, but they felt that the effect was not the same. A similar problem was mentioned by García de Teresa (2022), who found that, when mushrooms cannot be sold fresh, they were kept dried or in honey, but the Mazatecs considered that they lost their potency. For this reason, in the study area, the use of mushrooms for divinatory or medicinal purposes was limited to the rainy season, mainly in the month of July.

### ﻿Perceptions about mushrooms

Within the hot and cold classification system that exists in Mesoamerica, in El Peral, the inhabitants considered that Nanacatito is hot. For this reason, when you eat it, you should not go out in the open because you can get “*el aire*” (the air), a cultural illness believed to arise from exposure to cold air (Anonymous 16, female, 92 years old, 2024). This is something similar to what happens with the Otomí of Acambay (Estrada-Torres and Aroche 1987), who believe that some poisonous mushrooms can be used medicinally because they are hot entities. However, it is different with the Chinantecs from Oaxaca (López-García et al. 2020) and Tsotsiles from Chiapas (Ruan-Soto 2018), who consider all mushrooms cold, so, to counteract mushroom poisoning, hot elements such as pox (spirits produced by the distillation of fermented corn) must be consumed.

When inhabitants of El Peral talked about the divinatory mushrooms, they attributed human characteristics to them, such as feelings, like other Mexican indigenous groups, such as the Wixaritari of Jalisco do with all mushrooms (Haro-Luna et al. 2019). In El Peral, they talked about them as if they were a person. For example, they mentioned that “mushrooms feel” and “the mushroom is very smart” (Anonymous 10, female, 45 years old, 2022). For them, the mushroom is the one that talks to you and dictates the remedies or answers you are looking for. They can even get angry with you if you do things wrong. Therefore, “before consuming these mushrooms, you should ask their permission and talk to them” (Anonymous 15, female, 87 years old, 2023). Traditionally, before consuming them, the sick person or consumer engages in a loud conversation with the mushrooms, asking them about their concerns, while smoking them with copal (resin of *Burserabipinnata*). This practice has also been reported amongst the Chatinos (López-García et al. 2024), Mixes (Miller 1966) and Nahuas (Spiers et al. 2024), while amongst the Mazatecs, Catholic prayers are also incorporated into the ritual (Fagetti and Mercadillo 2022).

### ﻿Transmission of knowledge

In the study site, all members of the family unit, from children to the elderly, were aware of the optimal locations for the collection of these mushrooms and the corresponding seasonal patterns. Furthermore, they were aware of the traditional method of consumption, with mothers and fathers serving as the primary transmitters of this knowledge, as observed in other regions of Mexico (Garibay-Orijel et al. 2012; Haro-Luna et al. 2019). Grandparents were also involved, although this occurred less frequently. As is the case with other Mexican ethnic groups (Haro-Luna et al. 2022b), children in this community were taught to recognise and identify mushrooms at an early age. This was accomplished through practical observation, with children accompanying their parents to forests and streams to search for mushrooms. Similarly, children’s involvement in mushroom-related practices extends beyond identifying and collecting mushroom for food; they also play a role in the use of sacred mushrooms in traditional healing and divination. In particular, it is customary to send children to gather mushrooms on behalf of a sick person. Reflecting the seasonal availability of mushrooms, it is common to see children collecting them after school in August and selling them later.

Regarding consumption, the age at which individuals acquired the knowledge and skills associated with consumption practices varied considerably. Some individuals recalled that, between the ages of 14 or 19, their parents demonstrated the traditional process of consumption. In the interviews, they agreed that this variation is mainly due to the fear that young people feel about consuming mushrooms, even though they had observed adults doing so from a very young age. Others indicated that they acquired this knowledge only as adults, citing the need to seek guidance from their parents, grandparents or other community members with extensive knowledge on the subject. Four of the interviewees, aged between 60 and 95, recalled that individuals from other municipalities, such as San Mateo Mixtepec, sought the assistance of their parents in seeking to be cured using mushrooms. In this way, although with fear, they also learned by observing the rituals being performed.

According to what the authors observed and what was expressed by the interviewees, this knowledge is no longer transmitted when a family member changes religion. In the words of the people “there is a religion that no longer believes, they no longer eat it. It (mushroom) only speaks to those who believe it” (Anonymous 16, female, 92 years old, 2024). A similar phenomenon can be observed in Latin American countries regarding traditional knowledge. In recent years, social and economic changes in Latin America have caused marginalised sectors to change religion as a defence mechanism against poverty, loss of traditional values and moral norms (Andrade 2004). In Mexico, the incorporation of people with indigenous affiliation into non-Catholic denominations has led to the loss of traditional knowledge and original languages (Gutiérrez Portillo 2018). This also puts an end to traditional practices, leading to a shift in worldview and the way the world is understood (Cano-Contreras et al. 2009).

### ﻿Traditional uses of *Psilocybezapotecorum*

*Psilocybezapotecorum* mushrooms are mainly used for divinatory purposes, to predict the future or, as people said, “to know something”. They can also be used to remember things or events and as medicine to cure various physical and spiritual illnesses. The traditional use of this mushroom by the Zapotecs was previously reported in the undergraduate thesis of Amaya Pérez (2020). She mentioned that, in San Miguel Mixtepec, belonging to the Valles Centrales Region, they consumed *P.zapotecorum* to treat illnesses or to consult it for very difficult problems to solve, both family and social. This divinatory use is also present amongst the Mixes (Miller 1966) and Chinantecs (Rubel and Gettelfinger-Krejci 1976), as well as in the healing-divinatory practices amongst the Chatinos (López-García et al. 2024) and Mazatecs (Guzmán 2008; Estrada Martínez et al. 2016; Fagetti and Mercadillo 2022). On the other hand, this conceptualisation is unlike other indigenous groups in Mexico such as the P’urepechas or Wixaritari, who consider these mushrooms as toxic (González-Rivadeneira and Argueta 2018; Haro-Luna et al. 2019).

The main objective behind consuming these mushrooms is to obtain guidance or counsel regarding crucial decisions that have the potential to significantly impact an individual’s future. For example, in light of the considerable influx of migrants in recent times (Luis-García et al. 2022), one of the most frequently sought-after insights from Nanacates pertains to the feasibility of crossing the border for employment purposes. “Before you go to the United States, the mushroom tells you whether you are going to pass or not” (Anonymous 9, female, 50 years old, 2022). Similarly, there are accounts of women who enquired of mushrooms about the fidelity of their husbands who resided in the United States. “I presented the leaf (a dose of mushrooms wrapped in a leaf) to the woman from San Lorenzo to ascertain whether her husband would return from the north. He did not return, as he had formed an attachment with another woman there” (Anonymous 16, female, 92 years old, 2024). Additionally, consultations were recorded regarding landownership disputes. This illustrates how the practice of mushroom consultation adapts to the new socio-economic realities faced by communities, revealing how such traditions continue to address the changing needs of individuals.

The interviewees considered that mushrooms can diagnose illnesses and, during the trance caused by their consumption, they could see the causes of the illness. “It is like a medicine, it is a cure, it tells you what illness you are suffering from or why that happens, where it went wrong and why there is illness” (Anonymous 2, female, 63 years old, 2022). Within the Zapotec cosmovision (León 2003) and according to what was expressed by the people of El Peral, illness is considered a punishment for a mistake they made in the past or for an evil desired by a third person: “When someone hurts you, the mushroom tells you who is behind it. That’s why you feel that way, that person holds a grudge against you. It (the mushroom) will tell you everything” (Anonymous 15, female, 87 years old, 2023).

They were also used to cure culturally affiliated illnesses, common in Mesoamerican cultures (Idoyaga 2006; Campos-Navarro and Fagetti 2022), such as sadness, fright, the evil eye or lovesickness and they reveal who wished harm upon them or their loved ones, particularly children: “The *Nanacate* cures everything, like the evil eye. If someone does you harm, it heals you; it also helps with lovesickness, telling you whether you are loved or not” (Anonymous 9, female, 50 years old, 2022). The use of *Psilocybe* species to cure these types of illnesses was also reported in the Mazatec culture, where María Sabina explained to Estrada that, when someone is ill, it is because their spirit has fallen ill and it is the mushrooms or Niños Santos that heal the wounds of the spirit (Campos-Navarro and Fagetti 2022).

In this locality, Ni’to be’ya could also be used to learn how to heal physical ailments. It was described that, during the trance, the mushroom shows where and how to massage the body to heal it: “The mushroom teaches you if you are going to be a healer because it says ‘let me see your arm,’ and it massages you and shows you how to heal it” (Anonymous 15, female, 87 years old, 2023). A similar case was reported in western Mexico amongst the Wixarika ethnic group, where mushrooms were consumed to acquire shamanic abilities (Haro-Luna et al. 2019).

### ﻿Dose

In the community, it was believed that the quantity of mushrooms a person requires for a consultation depends on the amount of mezcal they need to drink to become drunk. For example, if someone is used to drinking heavily and can consume large amounts of mezcal, they will need a greater number of mushrooms to feel the effects. However, caution regarding excessive consumption is emphasised; it was mentioned that one should not eat too many. This was also reported by Miller (1966) in Mixe communities. In El Peral, when people compared mushrooms to alcohol, both were noted to be useful, but excessive use can turn into a vice. Both alcohol and mushrooms can be used as medicine, but their excessive or improper use may lead to problems. Therefore, the recommended dose ranged from three to five mushroom sporomes, depending on their size and the individual experience of the person. As already mentioned above, this differs from what has been reported amongst the Mazatecs, who consume approximately six pairs or 12 basidiomes (Guzmán 2008, 2014) and from the Mixes, who also consume mushrooms in pairs (Miller 1966).

Regarding consumption frequency, some people stated that mushrooms can only be eaten once a year, while others mentioned that it can be done several times, as many as necessary, provided the person is willing and the patient or consumer is not afraid. “You don’t eat them two or three times in the same year, just once” (Anonymous 11, female, 47 years old, 2022). “As much as you want and if you can handle it, you can eat them many times as long as you’re not afraid. If you buy three leaves, that means three times” (Anonymous 16, female, 92 years old, 2024). However, they believe that, if a person is very ill, the illness will resist the effects of the mushrooms, requiring them to consume a larger quantity or take them repeatedly.

### ﻿Traditional use methods

Once the Zapotecs of El Peral have obtained the mushrooms, either purchased or collected by themselves, they wait until nightfall to consume them. This is because they wait for the silence of the night to avoid interruptions and to be able to clearly hear what the mushroom “tells” them in peace. “They are eaten at night, in the silence, because with noise you can’t hear what they say” (Anonymous 8, female, 67 years old, 2022). This nocturnal consumption is generally the rule and had already been reported amongst the Chinantecs of Santiago Comaltepec, Oaxaca (Rubel and Gettelfinger-Krejci 1976), the Matlatzincs of San Francisco Oxtotilpan, State of Mexico (Guzmán and López-González 1970), the Mazatecs (Estrada Martinez et al. 2016; García de Teresa 2022; Fagetti 2023) and the Mixes of Mazatlán and Coatlán, Oaxaca (Miller 1966).

**Figure 5.**
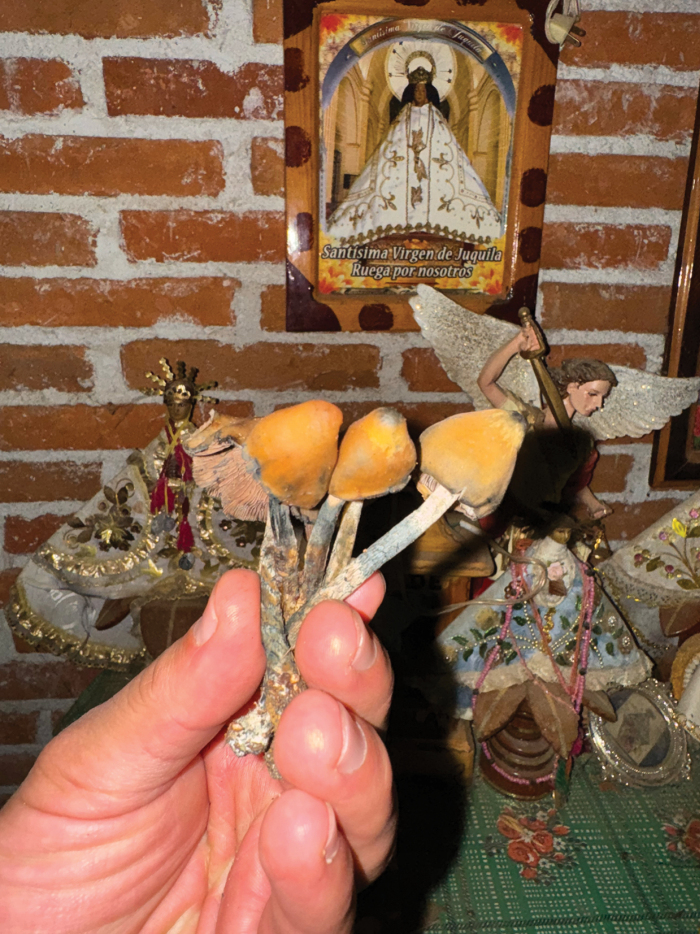
Dose of *Psilocybezapotecorum* ready to be incensed with copal (resin of *Burserabipinnata*) in front of the altar of San Miguel Arcángel.

Before consuming them, the basidiomes are incensed with copal (Fig. 5). This aligns with what has been reported in Mazatec “*veladas*” (Fagetti and Mercadillo 2022). The practice of burning copal has been carried out in various ceremonies performed by Mexican indigenous groups, particularly Mesoamerican ones, since before the Spanish conquest. According to Montúfar López (2016), in the indigenous worldview, this act strengthens the bond between humans and divine entities. Therefore, after the smoke, following the consumption of the mushrooms, a conversation with God or the divine will take place to know the future, receive answers or alleviate ailments.

According to the testimonies collected, people considered that it is appropriate for children, who have pure souls, to smoke the mushrooms in front of an altar. These altars can be dedicated to any saint and it is common for each household to have one with several Catholic images and fresh flowers. The altars most frequently observed were dedicated to Saint Michael, the Archangel (Fig. 5). However, this does not influence the visions one may have. “At the table with the saint, you take the mushroom and ask for permission, explaining why the mushroom will be consumed” (Anonymous 15, female, 87 years old, 2023). “The children pass the smoke and you make the questions to mushrooms” (Anonymous 11, female, 47 years old, 2022).

Before consuming them, the mushrooms were asked for permission and talked to “just as if they were a person” (Anonymous 2, female, 63 years old, 2022), explaining the purpose of their consumption. “Before eating them you must ask permission so that they don’t scare you and you must tell them what you are going to consume them for” (Anonymous 15, female, 87 years old, 2023). Once this is done, when you start with the ingestion of the mushrooms, you should only chew them with the front teeth, not with the molars. They described the taste as sour and it is advised to chew them for a considerable time. This specific practice of chewing mushrooms only with the front teeth and for an extended period had not been documented in other cultural groups.

When consuming mushrooms, the individual usually requests the presence of a companion who must remain in another room, silent and quiet. This person is tasked with watching over the consumer and being available in case the consumer becomes frightened or feels the need to leave the house during the trance. Leaving the house is considered the worst possible outcome, as the community mentioned that under the influence of the mushrooms, the “*Chaneque*” (naughty spirit, with the appearance of a child) could lead them astray in the wilderness and they might never find their way back home. In contrast to what was reported by Guzmán (2008) with the Mazatecs, the interviewees reiterated that it is not necessary to have a guide or healer present during mushroom ingestion. This only happens when the individual is afraid or unaware of the practice.

The effects of mushrooms can last for several hours, during which one may experience visions, hear voices or even communicate with the mushrooms themselves. However, it is crucial to interpret these messages correctly. The person accompanying the individual consuming the mushrooms should also remember if the sick person or participant mentioned anything during the trance, as this can aid in interpreting the messages and also because, after the effects wear off, some details may be forgotten.

It is worth noting that some individuals avoid consuming mushrooms on certain days, such as Tuesdays and Fridays or on the 5^th^ and 25^th^ of the month. Others prefer to consume them on Wednesdays, Thursdays or Sundays. Although these responses were not consistent across all interviewees, they were frequently mentioned by older adults. Unlike what has been reported regarding the ritual consumption of *Psilocybe* mushrooms, where sexual abstinence, refraining from alcohol and fasting were required (Carod-Artal 2015), in El Peral, they mentioned that none of these practices was necessary.

### ﻿Effect

After consuming mushrooms, individuals described sensations of warmth, laughter or sadness and, sometimes, all emotions within the same session. This varies depending on the purpose of the mushroom consumption and the emotional state of the consumer. Amongst indigenous users, crying during mushroom consumption can be interpreted as a way to reveal negative actions causing harm, a phenomenon also documented by Estrada (1977) in the biography of María Sabina. For example, excessive crying is associated with intense personal experiences, such as the revelation of an impending loss of a loved one. “When they cry a lot, something is going to happen to them, they grieve” (Anonymous 15, female, 87 years old, 2023). From a Western biomedical perspective, *Psilocybe* mushrooms induce somatosensory alterations such as euphoria, dizziness, uncontrollable laughter and joy, visual distortions, anxiety, paranoia, headaches, nausea, vomiting, diarrhoea, muscle pain, chills and pupil dilation (Sharma et al. 2023).

In El Peral, Bernardita, 45 years old, described these sensations after mushroom ingestion as “emotional whirlwinds”, where one can feel shame, happiness and sadness simultaneously, while also displaying unpredictable behaviour such as laughing and crying without apparent reason. Others described the sensation that the mushrooms “massage” them internally, a feeling they cannot fully explain, but that brings a sense of well–being, particularly in relation to physical ailments. Some even report seeing in their visions the movements or exercises they need to perform to regain mobility in an injured body part or alleviate joint pain. Similar actions were also reported by Estrada (1977), where María Sabina described her sensations after consuming mushrooms as a mystical experience in which she heard voices from another world, akin to the voice of a guiding father. She also recounted that, after ingesting them, she experienced a profound state of joy, spontaneous weeping and visions in which she perceived the presence of spiritual beings.

In terms of physical reactions, individuals may experience falls, dancing, feeling intoxicated or even the urge to go into the forest. For this reason, the consumer must be locked in their room to prevent them from getting lost. When the process and trance unfold correctly, the consumer engages in a conversation with an entity, which interviewees assumed to be the mushrooms themselves. As they mentioned: “It depends on how the person is, the mushroom starts talking and tells them what illness they have, who is harming them and what type of remedy they can use” (Anonymous 15, female, 87 years old, 2023).

Visions of snakes or animals that people usually fear can indicate that the mushroom is not communicating in a positive way and these are assumed to be signs of malevolence, whether their own or inflicted by someone else: “Sometimes you see snakes when someone has done harm to you” (Anonymous 11, female, 47 years old, 2022). After such negative or frightening visions, some decide not to consume mushrooms again. Those interviewed emphasised that the fear they felt from visions of dangerous animals, monsters or even death itself led them to avoid future consumption. These accounts align with what was documented by Guzmán (1990), who recorded his own experience with mushroom consumption. In his account, despite remaining conscious, he initially felt fear upon perceiving a tentacle-like beam of light extending towards him and witnessing the transformation of his mushroom drier into a castle. He also experienced distress when he found himself trapped in a vision where climbing a staircase became an arduous task. However, his experience eventually became more pleasant, contrasting with some of the interviewees in this study, who interpreted negative visions as warnings and consequently decided to refrain from further mushroom use (Mata et al. 2016). This contrast suggests that, while some individuals may re-interpret their experiences as insightful or transformative, others perceive them as overwhelmingly negative.

Although bad experiences were uncommon, the community had various remedies to counteract the effects of mushrooms for individuals experiencing negative reactions during the trance. For example, the simplest method was to sprinkle water on the person’s face. One of the most mentioned traditional remedies was drinking milk; some people specified that it must be milk from an older cow for the remedy to be effective. Others mentioned that yogurt or even holy water could also be consumed. Additionally, there was a more elaborate antidote, where a portion of the mushroom’s stipe was set aside and submerged in a glass of water with a few drops of lemon or in milk and the liquid was then consumed. They also mentioned that “so that they don’t stay crazy”, that is, to prevent someone from remaining in a permanent trance, corn was burned and used to make “*atole*” (hot, thick drink made from cooked and ground corn), which should be drunk. This information had not been previously documented in literature, so it represents a contribution to the understanding of traditional knowledge associated with these mushrooms. However, similar information exists amongst the Chinantecs, who reduced the effects of these mushrooms by drinking orange leaf tea or mezcal (Rubel and Gettelfinger-Krejci 1976). It should be noted that these methods are not necessarily used to counteract bad experiences, but rather to diminish the general effects of the mushrooms.

### ﻿Precautions

Certain precautions must be taken before consuming mushrooms, as there are various stories of individuals who experienced drastic changes after consumption. For instance, the Zapotec people emphasised that the negative effects of mushrooms occur when they are not chewed exclusively with the incisors and the molars are used instead. Additionally, mushrooms should not be consumed alongside other potent substances such as alcohol. Like the Chinantecs (Rubel and Gettelfinger-Krejci 1976) and in contrast to the Mazatecs (Estrada 1977), they do not deem sexual continence necessary prior to mushroom consumption.

Women should not consume mushrooms when they are nearing menopause. There was a case of a woman in the community who consumed them during that time, so it was believed that this caused her to lose her mind: “My aunt ate a mushroom and went crazy. But it was because she was about to go through menopause and it all came together, so it wasn’t the mushroom, it was the menopause that made her crazy” (Anonymous 12, female, 36 years old, 2022).

As mentioned above, there should always be someone watching over the consumer, especially to prevent them from leaving the house and getting lost while unconscious. However, they must also listen carefully to the instructions given by the mushroom, as there are accounts in which the mushroom indicates that the consumer should not be accompanied. In such cases, the attendant is informed and must leave the room or the house. This is similar in part to the Chinantecs of the community of Santiago Comaltepec, who consume mushrooms away from noise and any other human presence (Rubel and Gettelfinger-Krejci 1976).

Interviewees asserted that people who are not ill or do not have a need for consultation become addicted and suffer severe discomfort. Furthermore, if you are a bad person or have done bad things, the mushroom scolds you, showing the evil inside you and causing you to physically feel that evil within yourself.

### ﻿Use for children and pregnant women

Female informants confirmed that pregnant women, those who are breastfeeding and children can consume Nanacatitos. For example, in cases where a breastfeeding woman needs to consume mushrooms to heal or divine something personal or related to the baby, the effect does not pass to the infant through breast milk, although some mentioned that it can “make the baby act funny” (Anonymous 3, female, 78 years old, 2022). Additionally, mushrooms can be given to children if they are sick, though this does not always work, especially if they are very young, such as children under 10 years old. It is more common for the mother to consume the mushrooms herself in order to understand what is happening to her child and how to heal them. This contrasts with the perceptions of the Mixes, who believe that a pregnant woman should not consume mushrooms, as they considered them harmful for both the mother and the unborn child (Miller 1966).

### ﻿Use by new generations

Interviewees agreed that the traditional consumption of Nanacatitos persists only amongst adults and elderly people. They believe that young people no longer use them in the same way, nor do they believe in their ability to heal or predict the future. There was a widespread perception that young people use mushrooms without the necessary seriousness or respect, which, according to the interviewees, prevents them from obtaining medicinal or spiritual benefits. As one of them mentioned: “For those who have too much fun with it, it no longer speaks well” (Anonymous 16, female, 92 years old, 2024). This is because, when young people consume mushrooms, they do so for recreational purposes, particularly those who are not part of the community or the Zapotec people. This sentiment aligns with what has been reported by other authors (e.g. Estrada (1977); Letcher (2007); Guzmán (2011, 2014)), who pointed out that mushrooms have been commercialised and their use has lost the respect for ancestral practices.

As previously mentioned, this was confirmed by the testimonies of younger interviewees, aged between 16 and 22, who stated that they consumed mushrooms to feel or see things, but not in the way the elders described, without following any ritual, at any time of the day and in any specific location. On the other hand, it is worth noting that one of them was made to consume mushrooms by his mother to heal a contracture in his arm. During the effects of the mushrooms, he was able to see the exercises he needed to perform to recover and stop feeling pain. However, despite this, he expressed that other perceptions regarding the divinatory and medicinal properties of the mushrooms were beliefs of the “*abuelos*” (grandparents), as the elders of the village are called.

However, the use of mushrooms by outsiders is not frowned upon. People described it as “God places mushrooms in the mountains so that everyone can use them” (Anonymous 6, female, 40 years old, 2022). Therefore, they have no issue with foreigners consuming them. Nevertheless, mushrooms were seen as potentially dangerous if not used correctly, particularly amongst young people, who now use them as a recreational drug rather than as a medicine. We cannot assert that, in this case, they are considered toxic mushrooms; a more in-depth study, focusing on traditional classification, would be necessary to determine this. They associate inappropriate consumption with discomfort, as hallucinations and dizziness that do not convey a message or relief are perceived as distressing symptoms. For this reason, the community emphasised the importance of consulting the elders, who possess greater knowledge and expressed concern about the lack of this consultation and respect for tradition amongst the youth. As they mentioned, “young people no longer consult, they no longer believe and, because of that, all of this will disappear” (Anonymous 16, female, 92 years old, 2024).

## ﻿Final considerations

The diversity of names given to *Psilocybezapotecorum* in the community of El Peral, as well as the descriptions of its morphological characteristics, are evidence of different historical events and the transmission of traditional knowledge. Likewise, the respectful language used when referring to these mushrooms suggests that the relationship between the community and these mushrooms goes beyond mere functionality, placing them in an important healing space. In addition, their main use is related to their divinatory properties, being used to consult about the future and to solve personal and health problems.

According to what was documented in the interviews conducted, people were aware of the influence of climate change and deforestation on the availability of *Psilocybezapotecorum* basidiomes. The perception that the climate has changed adversely indicates a concern for the sustainability of natural resources and the impact of human intervention on the environment. This is especially true as mushrooms are a necessary resource for the treatment of certain health conditions and even a source of income for some families. Moreover, the decline in the abundance and availability of this resource has heightened awareness within the community, reinforcing the need to protect traditional knowledge and practices. Likewise, in the community of El Peral, there is a clear awareness of the possibility that the tradition of eating mushrooms is in danger of disappearing, mainly due to the growing disinterest of the new generations.

The influence of the mushroom picker’s emotional state and personal characteristics on the consumption experience revealed a belief system with a social and spiritual dimension. This is because, for example, the collector must be emotionally well, even joyful or happy when collecting the mushrooms and should not be violent or exhibit negative traits. If the collector is in a negative emotional state, the consumer may feel this during the trance, which could result in a bad experience. This belief is rooted in the idea that the emotional energy of the collector can affect the person consuming the mushrooms, potentially influencing the outcome of the experience. It also strengthens community cohesion, as these practices are not only individual, but deeply embedded in collective rituals and responsibilities. The involvement of children in the collection and their protagonism in this ritualised practice is also crucial for the transmission of knowledge between generations.

For the Zapotecs of El Peral, mushrooms have feelings and are perceived as beings capable of communicating with the consumer, reflecting their profound importance in local culture. The visions and emotions that mushrooms evoke were interpreted as divine messages, revealing illnesses as well as spiritual or physical conflicts, underlining the complex interaction between the consumer and the mushroom. This spiritual relationship calls for moderation and responsibility in their traditional use, as the experiences derived from their consumption play a crucial role in the individual’s perception of reality and spiritual health.

Although the inhabitants of El Peral were aware of the phenomena occurring in other parts of Oaxaca, such as Huautla de Jiménez and San José del Pacífico, where *Psilocybe* mushrooms have gained economic significance, in El Peral, their sale is conducted on a small scale and at very low prices. Even when the mushrooms are taken to larger towns like San Miguel Mixtepec or San Antonino El Alto, they were sold to those who request them as “medicine” to treat ailments that conventional medicine has been unable to resolve. This is because “psychedelic tourism” has not reached this area and it seems that, in the Zapotec Region, it has been concentrated only in San José del Pacífico, a passing town that belongs to San Mateo Río Hondo in the Sierra Sur Region, the same region where San Agustín Loxicha is located.

It is important to note that, in the case of El Peral, tourism is unlikely to arrive, at least in the coming years, due to the community’s strong sense of privacy and reserve. While, as mentioned in the results, community members believed that *Psilocybe* mushrooms can be used by anyone who needs them to address a problem, they are unwilling to welcome tourism of any kind in the area. An example of this is the experience of one of us (JAP), who attempted to organise wild mushroom exhibitions, but was denied permission by traditional authorities, who felt the community lacked the capacity and infrastructure to receive visitors. In 2024, JAP was able to hold an exhibition in the nearby town of San Miguel Mixtepec, but its primary purpose was to educate residents about the fungal biodiversity present in their forests.

However, the intergenerational gap is leading to a change in the use of mushrooms. Young people were using mushrooms for recreational purposes and, according to local perceptions, without the necessary respect for ancestral traditions. This has raised growing concerns amongst older adults, who fear that traditional practices and knowledge will be lost over time. Although mushrooms were seen as a divine gift available to all, both outsiders and community members, they considered that their misuse could be dangerous. In this sense, the commercialisation of mushrooms and their use without adult guidance not only dilutes their healing and divinatory powers, but also threatens the cultural heritage passed down through generations.

Although the Nanacates are traditionally used for divinatory and medicinal purposes, their consumption without proper knowledge or guidance can pose health risks. Potential dangers include misidentification and, therefore, confusion with potentially toxic species, adverse physiological reactions and psychological effects that may be distressing. It is essential to recognise that the perceptions and experiences associated with mushroom consumption amongst the people of El Peral are deeply rooted in their cultural framework and cosmovision. The meanings and insights derived from these experiences cannot be separated from the local knowledge systems and spiritual beliefs that shape them, highlighting the need for a culturally contextualised understanding of their use.

This is the first formal record of the use of hallucinogenic mushroom amongst Zapotecs of the Valles Centrales Region in Oaxaca. Additionally, it should be noted that the community, while agreeing with the use of mushrooms by outsiders and recognising the current existing projects for the widespread use of these mushrooms, emphasises that they should be used for healing or curative purposes, not as a form of entertainment. Rather than imposing from a Western vision what we think is “best” for the communities, a patriarchal attitude should be avoided and the cultural autonomy of these groups recognised. Respecting and valuing their practices require asking them directly how they perceive these issues and how they want their ancestral knowledge to be transmitted and preserved, without imposing external models that could distort their traditions.

We would like to emphasise that the people of El Peral demonstrated consistent enthusiasm in sharing their knowledge about mushroom use, particularly upon understanding that this was a formal research project. This enthusiasm was evident during the interviews and the two meetings convened by local authorities. Notably, discussions about Nanacates were often more fluid than those related to edible mushrooms. As previously mentioned, community members perceived that this traditional knowledge is fading and they viewed this study as a valuable opportunity to document their practices. This also underscores the importance of adhering to ethical guidelines, also being transparent at all times about the scope and objectives of the research, as well as the capabilities and limitations of the researchers. Maintaining honesty fosters trust, strengthens relationships with participants and facilitates collaboration with traditional authorities and the research team towards a shared goal. However, it is important to acknowledge that we cannot assume unanimous agreement within the community, nor can we predict whether those interviewed would adopt the same attitude towards other visitors or researchers in the future.

## ﻿Additional information

### Adherence to national and international regulations

The data presented were collected with the consent of the interviewees and local authorities. They also agreed to its publication for scientific purposes and to contribute to the preservation of their knowledge. The code of ethics of the Latin American Society of Ethnobiology was followed (Cano-Contreras et al. 2016).

### Author ORCIDs

Mara Ximena Haro-Luna https://orcid.org/0000-0002-3286-1038

Felipe Ruan-Soto https://orcid.org/0000-0002-2476-027X

Virginia Ramírez-Cruz https://orcid.org/0000-0002-5197-1304

Laura Guzmán-Dávalos https://orcid.org/0000-0002-4390-3678

### Data availability

The datasets used and analyzed during the current study are available from

the corresponding author on reasonable request.
